# Diverging lives after death: Differences in loneliness in widowhood across race in the United States

**DOI:** 10.1093/geroni/igaf122.3572

**Published:** 2025-12-31

**Authors:** Micah Tan

**Affiliations:** Princeton University, Princeton, New Jersey, United States

## Abstract

While racial heterogeneity in the widowhood effect on mortality has been explored, heterogeneous effects on social outcomes like loneliness across race in the United States have yet to be investigated. Using a sample of widow(er)s from the Health and Retirement Study (HRS) aged 50 and above (n = 4,835), this study addresses this gap by examining if the impact of widowhood on loneliness varies across black and white widow(er)s. An event study approach with two-way fixed effects models is employed to examine how the likelihood of feeling lonely changes after widowhood for black and white widow(er)s. Results suggest that white respondents experience a stronger widowhood effect on loneliness than black respondents, and that group-level differences across race are driven by differences among widow(er)s living alone with differences in the widowhood effect on loneliness across race being statistically insignificant among those living with others. Differences in the availability of proximate social resources and in the level of stigma experienced after widowhood are argued to explain heterogeneous effects across race, with black widow(er)s having greater access to proximate social resources and experiencing less stigma in widowhood. Preliminary tests find evidence in support of the mediating effects of these mechanisms in explaining racial differences in the widowhood effect on loneliness. These findings shed light on why racial disparities in well-being diminish in later life, with transitions like widowhood making social resources that black Americans have greater access to more important for well-being, thus extending existing life course perspectives on racial disparities in well-being.

